# Adult Left Liver Transplantation With Mesocaval Shunt With Porto‐Mesenteric Disconnection: Long‐Term Outcome

**DOI:** 10.1111/ctr.70292

**Published:** 2025-08-28

**Authors:** Jérôme Dumortier, Sophie Chopinet, Romain L'Huillier, Serban Puia‐Negulescu, Mustapha Adham, Laurent Milot, Olivier Boillot

**Affiliations:** ^1^ Department of Digestive Diseases Hôpital Edouard Herriot Hospices Civils De Lyon Lyon France; ^2^ Université Claude Bernard Lyon 1 Lyon France; ^3^ Department of Digestive Surgery and Liver Transplantation Hôpital La Timone Assistance Publique‐Hôpitaux De Marseille Marseille France; ^4^ Aix Marseille University, LIIE Marseille France; ^5^ Aix Marseille University, CERIMED Marseille France; ^6^ Department of Radiology Hôpital Edouard Herriot Hospices Civils De Lyon Lyon France

**Keywords:** liver transplantation, portal vein inflow modulation, small‐for‐size grafts, survival

## Abstract

Aiming to decrease portal venous pressure and to minimize the risk of small‐for‐size syndrome when using a partial liver graft for liver transplantation (LT), surgical techniques modulating venous portal inflow have been proposed. We report here our experience on the long‐term outcome after adult left split LT with mesocaval shunt (MCS) with porto‐mesenteric disconnection (PMD). Between March 1996 and March 2010, 33 adult patients underwent LT from a full‐right/full‐left SLT for two adult recipients; portal vein inflow modulation through a MCS with PMD was realized in 10 cases. The study population consisted of 13 left liver and 15 right liver recipients who survived more than 1 year without retransplantation. The technique of mesenterico‐portal disconnection with MCS which allowed to decrease the mean portal vein pressure to 14.2 mm Hg, was applied in six recipients who survived more than 1 year without retransplantation: five males and one female, with a mean age of 60 years (range: 53–66) who all received a left split graft, with a mean weight and GRWR of 562 g (range: 430–740) and 0.79% (range: 0.61–0.98), respectively. During follow‐up, four patients with MCS (4/6) experienced hyperammonemic encephalopathy, after a mean delay of 9.1 ± 7.9 years after LT (ranging 2.6–20.1). The outcome was favorable with symptomatic treatment with lactulose ± rifaximin in all cases except one, who underwent surgical closure of the shunt. In conclusion, our results emphasize that portal vein inflow modulation through MCS during LT can be complicated by late occurrence of hyperammonemic encephalopathy.

**Abbreviations**
GRWR: graft‐to‐recipient weight ratio
INR: international normalized ratio
LT: liver transplantation
MCS: mesocaval shunt
MELD: model for end‐stage liver disease
PMD: porto‐mesenteric disconnection
PVP: portal vein pressure
SFSS: small‐for‐size syndrome

## 1 Introduction

In an era of organ scarcity, ways to increase the number of available liver grafts for liver transplantation (LT) include domino LT, living donor LT (LDLT), split LT (SLT), and circulatory death donors LT. Full‐right‐full‐left SLT for two adult recipients is globally rarely conducted. Indeed, left LT for adult recipients is still a matter of debate because of uncertain graft and patient outcomes related to a small liver mass; small‐for‐size syndrome (SFSS) and technical failures are the main causes of early graft loss [1, 2]. Nevertheless, technical refinements, more accurate recipient selection, and better donor/recipient matching led to improved results of left LT in adult recipients [3]. Graft size, occurrence of SFSS, elevated portal vein pressure (PVP), recipient status, and donor age were found to be prognostic factors for recipient and graft outcomes when a small‐for‐size liver graft is transplanted in adult recipients [4]. A small‐for‐size liver graft usually corresponds to a graft volume of less than 0.8% of the graft‐to‐recipient body weight ratio (GRWR), which approximately corresponds to 40% of an adult recipient's standard liver volume [5, 6, 7]. Aiming to decrease PVP and to minimize the risk for SFSS, surgical techniques modulating venous portal inflow have been proposed, including splenic artery ligation, splenectomy, or portosystemic shunts [4]. The present study aimed to report the long‐term outcome after adult left split LT with mesocaval shunt with porto‐mesenteric disconnection (MCS with PMD), according to the surgical procedure we first described in 2002 [8].

## 2 Patients and Methods

### 2.1 Study Design

Between March 1996 and March 2010, 33 adult patients underwent LT from a full‐right full‐left SLT for two adult recipients: 17 left livers and 16 right livers (one right liver was not used secondary to a large hematoma). Long‐term outcome was analyzed for all recipients who survived more than 1 year after LT without retransplantation. This study was conducted in accordance with both the Declaration of Helsinki and Istanbul, and no donor organs were obtained from executed prisoners or other institutionalized persons. According to French law (Loi Jardé), retrospective studies do not require Institutional Review Board (IRB) approval. Our center closed at the end of 2010 due to local reorganization within the Hospices Civils de Lyon.

### 2.2 Graft and Donor Selection

**FIGURE 1:**
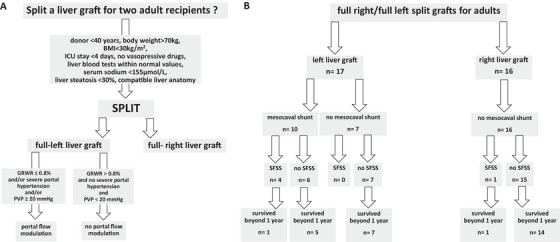
Split liver transplantation for two adults. (A) Decision‐making algorithm for split liver transplantation for two adults. (B) Flowchart of the patients included in the study.

Donor selection criteria for splitting a cadaveric liver for two adult recipients were as follows: age less than 40 years, stay in intensive care unit less than 4 days, absence or low doses of vasopressive drugs, liver blood tests within normal values, serum sodium below 155 mmol/L, liver steatosis less than 30%, and body weight over 70 kg with a body mass index <30 kg/m^2^. Our policy was to try to allocate to a given recipient a left liver with a GRWR over 0.8% if possible; however, in case of a predicted low GRWR (below 0.8%) and/or presence of severe decompensated portal hypertension (ascites, encephalopathy) and/or intra‐operative high PVP (≥20 mm Hg), the indication for MCS with PMD procedure was considered on a case‐by‐case basis, and may be performed during the surgical procedure of LT (Figure 1A).

### 2.3 Surgical Procedures

All 17 splitting procedures of cadaveric liver grafts were performed ex situ on the back table. In all cases, the left graft comprised segments I–IV, the middle hepatic vein, and the inferior vena cava. The common portal, arterial, and biliary elements were usually left to the right hemi‐liver unless a specific need for the left liver was mandatory. Hemostasis of the cut surface was secured by specific vessel ligations and running 5/0 sutures covering the whole surface. Iliac veins were harvested in all donors.

**FIGURE 2:**
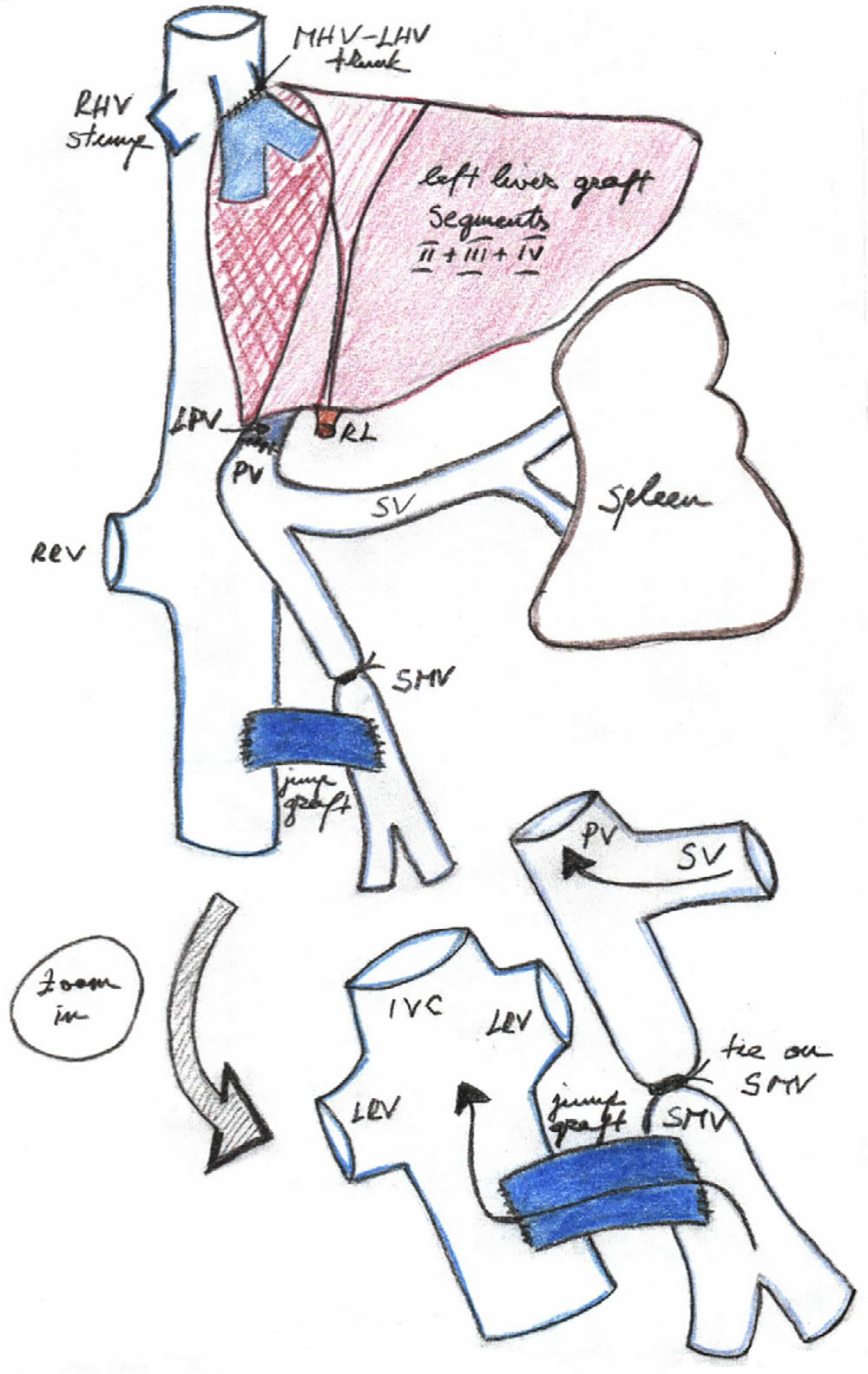
Adult liver transplantation with split left liver graft associated with mesocaval shunt with porto‐mesenteric disconnection. A mesocaval shunt is fashioned first using a donor iliac vein as a jump graft. A left liver graft is then implanted in the standard fashion. IVC, inferior vena cava; LHV, left hepatic vein; LPV, left portal vein; LRV, left renal vein; MHV, middle hepatic vein; PV, portal vein; RHV, right hepatic vein; RL, round ligament; RRV, right renal vein; SMV, superior mesenteric vein; SV, splenic vein.

Left liver SLT procedure consisted of recipient total hepatectomy with inferior vena cava resection or preservation according to the conditions; left and right vascular and biliary elements were divided into the hilum to keep enough length for the anastomoses with those of the graft. Graft implantation started with supra and infra‐IVC anastomoses with 4/0 and 5/0 running sutures, respectively, or a piggyback anastomosis in case of recipient IVC preservation. Then, the recipient portal vein (or its left branch) was anastomosed to the donor left portal vein with 6/0 running sutures, and the graft was reperfused. The recipient proper hepatic artery or one of its branches, depending on the size and quality, was anastomosed to the graft left hepatic artery by using 8/0 interrupted sutures. Duct‐to‐duct biliary anastomosis between the donor left hepatic duct and the recipient common hepatic duct with interrupted 6/0 resorbable stitches and external drainage was the gold standard technique whereas a hepaticojejunostomy was an alternative option. The left graft was implanted in a medial position secured by the placement of several stitches to avoid its rotation to the right. In case MCS with PMD was indicated, the first step of the surgery began with the completion of a meso‐caval shunt with interposition of the donor iliac vein, which was able to decrease portal hypertension and facilitate liver dissection. At the end of the transplant procedure, portal venous pressure was undertaken with and without MCS clamping; in case of elevated portal pressure over 20 mm Hg when clamping the MCS, it was left opened and downstream ligation of the superior mesenteric vein was performed (Figure 2) [8].

Right liver graft was implanted using the piggy‐back technique. End‐to‐end anastomoses were performed for the portal vein and hepatic artery, while duct‐to‐duct anastomosis without T‐tube was the standard for the biliary anastomosis, with hepaticojejunostomy as an alternative.

Finally, for all grafts, portal vein pressure was taken by direct needle puncture, ultrasound examination with Doppler was carried out, a cholangiography was performed through the biliary drainage when present and a liver biopsy was taken at the time of wound closure.

### 2.4 Definition of Small‐for‐Size Syndrome

SFSS was defined according to Soejima et al. [9], based on hyperbilirubinemia and ascites 14 days after LT with a partial liver graft, and after exclusion of technical complications (e.g., arterial or portal occlusion, outflow congestion, biliary leak), immunological (e.g., rejection), and infectious (e.g., cholangitis, sepsis) events.

### 2.5 Immunosuppression and Postoperative Care

The initial immunosuppressive regimen was based on a calcineurin‐inhibitor: cyclosporin or tacrolimus. Induction therapy by anti‐interleukin‐2 receptor or polyclonal antibodies was mainly administered in case of acute kidney injury. Starting on postoperative Day 1, corticosteroids were tapered down to reach a maintenance dose of 0–5 mg/day at 6 months post‐transplantation. Azathioprine, mycophenolate mofetil and sirolimus/everolimus were either administered as part of an initial triple immunosuppressive regimen or introduced during follow‐up as a maintenance immunosuppressive agent. Follow‐up visits were ensured every 3–12 months after first year post‐LT. Occurrence of surgical and medical complications was systematically recorded.

A CT scan and a liver biopsy were systematically realized 1 year after LT, at 5 years, and every 5 years thereafter. Graft volume measurement was performed and we reported the last one available.

### 2.6 Statistical Analysis

Descriptive statistics were used to summarize the baseline characteristics of the study population. Continuous variables were expressed as means with standard deviations (SD), and categorical variables were presented as frequencies and percentages. Comparisons between the right liver and left liver graft groups were performed using the Student's *t*‐test for continuous variables and the Chi‐square test or Fisher's exact test for categorical variables, as appropriate. A *p* value of less than 0.05 was considered statistically significant for all analyses.

To assess survival outcomes, Kaplan–Meier survival curves were constructed for the overall survival analysis, stratified by graft type (right vs. left liver). An actuarial incidence of complications of MCS with PMD was built. The log‐rank test was used to compare survival curves between the groups. Statistical analyses were conducted using SPSS version 23.0 (IBM Corp, Armonk, NY), and all tests were two‐tailed.

## 3 Results

### 3.1 Study Population

From the 33 adult patients who underwent LT from full‐right/full‐left SLT, 1 (6.2%) patient died in the full‐right group while 4 (23.5%) patients died including 2 after retransplantation in the full‐left group within the first year in a mean delay of 25 days (range: 6–61). The causes of death were small‐for‐size graft non‐function in one case, necrosing enteritis in one case, sepsis after retransplantation in two cases for graft failure secondary to small‐for‐size graft non‐function, (these four cases in the left liver group, all had a MCS) and cardiac failure in one case (the only one in the right liver group). A SFSS occurred in one case in the right liver group, and in four cases in the left liver group (all had MCS with PMD); two patients survived without retransplantation (one in each group).

Therefore, the study population consisted of 13 left‐liver and 15 right‐liver recipients (Figure 1B). Study population characteristics according to the type of liver graft are summarized in Table 1. It included a majority of men transplanted for alcohol‐related liver disease. The comparison between the two groups (left vs. right liver grafts) disclosed that left livers were significantly smaller, more often given to a woman recipient, of lower body weight. There was no difference regarding liver function at the time of LT. Two patients (all with left liver) had a GRWR below 0.8% and all underwent MCS with PMD. At the last follow‐up, the difference in graft weight/volume had disappeared, due to a significantly greater volume increase of left livers (2.70 fold vs. 1.84 fold for right livers).

**TABLE 1:** Study population characteristics according to the type of liver graft.

	Right liver (*n* = 15)	Left liver (*n* = 13)	*p* value
Liver transplantation			
Gender (M/F)	14/1	7/6	**0.029**
Mean age (years)	53.6 ± 7.2	55.4 ± 12.8	0.832
Mean body weight (kg)	80.8 ± 14.0	61.2 ± 11.6	**0.001**
Mean total bilirubin (µmol/L)	67 ± 102	69 ± 134	0.773
Mean INR	1.64 ± 0.56	1.48 ± 0.36	0.604
Mean serum creatinine (µmol/L)	78 ± 15	80 ± 21	0.387
Mean MELD score	14 ± 8	15 ± 6	0.504
Child class C (%)	53.3	46.1	0.707
Liver disease (*n*)			0.747
Alcohol‐related liver disease	8	7	
Virus‐related cirrhosis	4	2	
Retransplantation	0	2	
Other	3	2	
*Associated hepatocellular carcinoma*	*3*	*3*	
Combined kidney transplantation	0	1	0.464
Donor age (years)	25.9 ± 7.2	24.8 ± 6.6	0.844
Donor gender (M/F)	13/2	10/3	0.639
Graft weight (g)	907 ± 211	591 ± 116	**0.001**
GRWR (%)	1.13 ± 0.21	0.99 ± 0.27	0.227
GRWR below 0.8% (*n*)	0	3	0.087
Portal vein inflow modulation (mesocaval shunt)	0	6	**0.005**
Cold ischemia time (min)	632 ± 150	682 ± 194	0.678
Last follow‐up			
Mean weight (kg)	76.1 ± 11.9	68.4 ± 15.3	0.094
Mean total bilirubin (µmol/L)	8 ± 3	10 ± 8	0.390
Mean INR	1.0 ± 0.0	1.1 ± 0.0	0.964
Normal liver function/injury blood tests (%)	73.3	84.6	0.654
Graft final volume (mL)	1650 ± 372	1524 ± 372	0.186
Graft volume (final/initial)	1.84 ± 0.28	2.70 ± 0.98	**0.038**
GRWR final (%)	2.21 ± 0.38	2.31 ± 0.80	0.878

*Note:* The bold values indicate statistically significant *p*‐values (*p* <0.05).

Abbreviations: GRWR, graft‐to‐recipient weight ratio; INR, international normalized ratio; MELD, model for end‐stage liver disease.

### 3.2 Survival and Long‐Term Outcome

**FIGURE 3:**
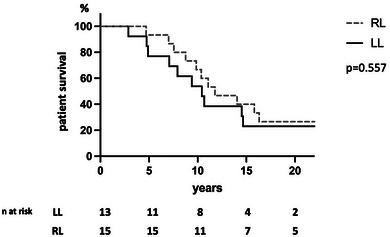
Actuarial patient and graft survival according to the type of liver graft (left vs. right): survival was 76.9% at 5 years, 60.0% at 10 years, 40.0% at 15 years, and 26.7% at 20 years for left grafts, versus 93.3% at 5 years, 53.8% at 10 years, 23.1% at 15 years, and 23.1% at 20 years for right grafts (*p* = 0.557).

Mean follow‐up after LT was 14.2 ± 6.0 years for the right liver group and 10.8 ± 5.7 years for the left liver group. After 1 year, no patient underwent retransplantation. Ten patients died in the right liver group (66.6%) and 11 in the left liver group (84.6%). No death was related to liver failure, and the main cause of death was de novo malignancies (38.1%). Patient and graft actuarial survival was similar regarding the type of graft (left vs. right) (Figure 3).

### 3.3 Inflow Modulation Group

The technique of MCS with PMD was applied in six recipients who survived more than 1 year without retransplantation: five males and one female, with a mean age of 60 years (range: 53–66) and a mean MELD score of 17 (range: 9–25) who received a left split graft from young donors (mean age: 25 years) with a mean weight and GRWR of 562 g (range: 430–740) and 0.87% (range: 0.61–0.98), respectively. The technique of MCS with PMD allowed a mean PVP drop of 7.6 mm Hg from 21.8 to 14.2 mm Hg.

**FIGURE 4:**
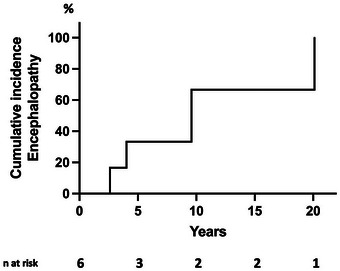
Actuarial occurrence of hyperammonemic encephalopathy after LT with left liver graft associated with MCS with PMD: 33.3% at 5 years, 66.6% at 10 years, 66.6% at 15 years, and 100.0% at 20 years.

During follow‐up, four patients with MCS with PMD (66.6%) experienced hyperammonemic encephalopathy, after a mean delay of 9.1 ± 7.9 years after LT (ranging 2.6–20.1) (Figure 4). The diagnosis of excessive mesenteric venous blood diversion through the shunt was made since all patients had a liver graft biopsy, which disclosed normal or subnormal parenchyma. Looking individually at each left liver recipient, with or without MCS, in only one case did we note the persistence of a probably insufficient hepatic volume associated with an alteration in hepatic function possibly due to persistent spontaneous porto‐systemic shunts that could explain the occurrence of hyperammonemic encephalopathy (Table 2). The outcome was favorable with symptomatic treatment with lactulose ± rifaximin in all cases except one. In this case, the MCS was surgically closed with restoration of the superior mesenteric venous flow to the graft.

**TABLE 2:** Characteristics of the 6 patients who underwent left liver LT with MCS with PMD and survived after 1 year.

Pt	Age at LT (years)	Gender	Initial GRWR (%)	PVP after clamping MCS (mmHg)	PVP after opening MCS^a^ (mmHg)	SFSS	hyperammonemic encephalopathy	Final GRWR (%)	Final prothrombin rate	Final total bilirubin (µmol/L)	Liver graft histology (time from LT)	Treatment of hyperammoniemia
1	66	F	0.61	19	11	No	No	3.36	66%	15	Recurrent HCV hepatitis METAVIR A1F1 (5 years)	−
2	58	M	0.89	26	15	No	Yes	2.83	80%	6	Normal (5 years)	Lactulose
3	59	M	0.98	27	17	No	Yes	2.21	65%	14	Mild rejection, no fibrosis (2 years)	Surgical revision
4	64	M	0.63	ND	ND	No	Yes	0.89	54%	37	Steatosis, no fibrosis (5 years)	Lactulose + rifaximin
5	60	M	0.81	17	13	No	Yes	1.88	85%	7	Steatosis, no fibrosis (10 years)	Lactulose + rifaximin
6	53	M	0.81	20	16	Yes	No	1.48	80%	9	Steatosis, no fibrosis (5 years)	−

Abbreviations: GRWR, graft‐to‐recipient weight ratio; LT, liver transplantation; MCS, mesocaval shunt; PVP, portal vein pressure; SFSS, small for size syndrome.

^a^ Mesocaval shunt opening and downstream superior mesenteric vein ligation; ND, not done;

## 4 Discussion

By now, using partial liver grafts (from a living or a deceased donor) is a major way to increase the number of available grafts, for both children and adults. Nevertheless, for adults, some partial grafts are not able to provide sufficient liver mass/volume and function. This led to the concept of SFSS in the early 2000's, even if the definition varies among studies. We used in the present study the simple definition of Soejima et al. in 2006 [9]. In 2017, a guideline was published by the International Liver Transplantation Society, encompassing three recommendations of strong evidence regarding on prevention of SFSS after LDLT [10]. The first was “graft injury and dysfunction in SFSS is not only a reflection of graft size but also of graft quality and degree of recipient portal hypertension causing graft hyperperfusion.” The second was “monitoring of the portal vein and hepatic artery hemodynamics is highly recommended for the early diagnosis, prevention and management of SFSS.” The third was “portal inflow modulation by splenic artery ligation/embolization or other portosystemic shunts is effective in the prevention and treatment of SFSS.” After the initial description of the procedure in 2002 [8], we report here the long‐term outcome after adult left split LT with MCS with PMD.

Portal vein inflow modulation by performing a portosystemic shunt has been proposed as a surgical option to reduce the incidence of SFSS but only a small number of cases have been reported by now [8, 11, 12, 13, 14, 15, 16, 17, 18, 19, 20]. This technique was usually applied to patients who received a graft with less than 0.8% of GRWR and/or with ≥20 mm Hg of PVP. No long‐term outcome has been reported by now, until our present report. We report that hyperammonemic encephalopathy occurred in more than half of the patients with MCS with PMD with normal or nearly normal liver function who survived more than 1 year (4/6), more than 20 years after LT in the latter case. In our patients, the outcome was favorable with symptomatic treatment with lactulose ± rifaximin in all cases except one; in this case, the MCS was surgically closed with restoration of the superior mesenteric venous flow to the graft. Therefore, our results suggest that the question of shunt closure away from the LT remains open. In the 2023 International Liver Transplantation Society, International Living Donor Liver Transplantation Group, and Liver Transplant Society of India Consensus Conference recommendations for the prevention of SFSS in LDLT, hemiportocaval shunt was recommended for surgical portal inflow modulation in exaggerated portal hypertension but it was stated that it may come at risk of portal steal and graft hypoperfusion (strength of recommendation: moderate; level of evidence: moderate) [21].

Other than a portosystemic shunt, portal venous flow can also be reduced by ligating the splenic artery. Ito et al. reported in 2003, seven cases (from a total series of 79) of adult LDLT with splenic artery ligation following arterial reperfusion, during which PVP was monitored [22]. For Days 2–4 and 9–11, recipients of small‐for‐size graft (<0.8% of body weight) displayed significantly higher PVP than recipients of larger grafts. Splenic artery ligation immediately reduced PVP from 10–20 mm Hg (median, 16 mm Hg) to 9–13 mm Hg (median, 11 mm Hg; *p* = 0.02). Posttransplant PVP was significantly lower in patients with splenic artery ligation than in patients without from Days 2 to 7 despite small graft size. Early PVP in splenic artery ligation patients was consistently below 20 mm Hg, and survival was significantly better than in patients without splenic artery ligation and with high early PVP (*p* < 0.01). Splenic artery ligation as the first line of surgical portal inflow modulation was recommended in the 2023 International Liver Transplantation Society, International Living Donor Liver Transplantation Group, and Liver Transplant Society of India Consensus Conference recommendations (strength of recommendation: strong; level of evidence: moderate) [21].

Combining a splenectomy to a LT could also be an effective option to modulate portal venous flow and overcome SFSS [23]. Yoshizumi et al. recently reported their large experience on simultaneous splenectomy on graft function and long‐term outcomes after LDLT [24]. These authors compared 258 recipients who underwent LT + splenectomy to 62 who underwent LT alone. In addition, to overcome selection bias, propensity score matching was performed (*n* = 50 in each group). In the entire population, recipients undergoing simultaneous splenectomy showed better early graft function, as well as lower sepsis frequency within 6 months after transplantation, and better graft survival. Propensity score matching analysis disclosed that recipients who underwent splenectomy had a lower frequency of early graft dysfunction, a lower frequency of SFSS, better early liver function (from bilirubin level and INR), a lower risk of sepsis within 6 months, and better graft survival. Univariate analysis revealed that not undergoing splenectomy (HR 3.06; 95% CI 1.07–11.0; *p* = 0.037) was the only risk factor for graft loss after LDLT. In addition, white blood cell and platelet counts increased significantly in the group of recipients with splenectomy. Such an effect cannot be expected after splenic artery ligation. In conclusion, the authors recommended simultaneous splenectomy in case a small graft (<35% of standard liver weight) is predicted preoperatively, or for patients with portal hypertension or high PVP (above 20 mm Hg) after reperfusion in LDLT. Nevertheless, it must be noted that the splenectomy is associated with a risk of portal vein thrombosis [25], probably low in the context of LT and resolution of portal hypertension. In the large cohort of Yoshizumi et al. it occurred in only 1.2% of the patients [24]. This low incidence was close in the series of Badawy et al. who compared 88 patients who underwent LDLT with concomitant splenectomy to 76 patients without [26]. The incidence of portal vein thrombosis was not different between groups, 5.7% (splenectomy) versus 2.6%. However, Kurata et al. and Linares et al. reported a greater incidence of 33% and 25%, respectively [27, 28]. In the 2023 International Liver Transplantation Society, International Living Donor Liver Transplantation Group, and Liver Transplant Society of India Consensus Conference recommendations, splenectomy was recommended as another effective modality of portal inflow modulation, but being associated with increased morbidity (strength of recommendation: moderate; level of evidence: moderate) [21].

Finally, pharmacologic intervention could be another method of portal flow modulation. Among the pharmacological agents, somatostatin or analogs are the most promising. Recently, Troisi et al. performed a randomized controlled trial for evaluating the impact of somatostatin for controlling portal hypertension after LT, and showed that somatostatin decreased the portal pressure by 29% despite no significant difference in survival with the control arm [29]. The Achilles’ heel of this treatment is its short duration (days) of action due to tachyphylaxis.

In conclusion, our results emphasize that portal vein inflow modulation through MCS with PMD is not always necessary in the context of adult left LT, can probably prevent the occurrence of SFSS in some cases, but can even be deleterious in some cases in the long‐term. Late occurrence of hyperammonemic encephalopathy can require medical treatment and, eventually, surgical reintervention.

## Conflicts of Interest

The authors declare no conflicts of interest.

## Data Availability

The data that support the findings of this study are available on request from the corresponding author. The data are not publicly available due to privacy or ethical restrictions.
